# Novel immune modulators used in hematology: impact on NK cells

**DOI:** 10.3389/fimmu.2012.00388

**Published:** 2013-01-03

**Authors:** Stephanie Krieg, Evelyn Ullrich

**Affiliations:** ^1^Hematology and Oncology, Department of Internal Medicine 5, University of Erlangen-NurembergErlangen, Germany; ^2^Laboratory for Cellular Immunology, Department of Pediatric Hematology and Oncology, Children’s Hospital, Goethe UniversityFrankfurt, Germany; ^3^Center for Cell and Gene Therapy, Goethe UniversityFrankfurt, Germany

**Keywords:** NK cell, IMiD^®^, TKI, Glivec^®^, bortezomib, thalidomide, lenalidomide, HDACi

## Abstract

There is a wide range of important pharmaceuticals used in treatment of cancer. Besides their known effects on tumor cells, there is growing evidence for modulation of the immune system. Immunomodulatory drugs (IMiDs^®^) play an important role in the treatment of patients with multiple myeloma or myelodysplastic syndrome and have already demonstrated antitumor, anti-angiogenic, and immunostimulating effects, in particular on natural killer (NK) cells. Tyrosine kinase inhibitors are directly targeting different kinases and are known to regulate effector NK cells and expression of NKG2D ligands (NKG2DLs) on tumor cells. Demethylating agents, histone deacetylases, and proteasome inhibitors interfere with the epigenetic regulation and protein degradation of malignant cells. There are first hints that these drugs also sensitize tumor cells to chemotherapy, radiation, and NK cell-mediated cytotoxicity by enhanced expression of TRAIL and NKG2DLs. However, these pharmaceuticals may also impair NK cell function in a dose- and time-dependent manner. In summary, this review provides an update on the effects of different novel molecules on the immune system focusing NK cells.

## INTRODUCTION/BACKGROUND

Immunomodulatory drugs (IMiDs^®^) have been introduced in the treatment of cancer with important clinical success. In addition to classical chemotherapeutic treatments, the novel classes of IMiDs^®^ have been developed for the treatment of multiple myeloma (MM), myelodysplastic syndrome (MDS), chronic lymphatic leukemia (CLL), and B cell lymphoma (Bartlett et al., 2004a).

Tyrosine kinase inhibitors (TKIs) are administered in patients with tumors overexpressing kinases like BCR/ABL, stem cell factor receptor (c-KIT), platelet-derived growth factor receptor (PDGFR) and vascular endothelial growth factor receptor (VEGFR). TKIs are used in first-line for chronic and acute myeloid leukemia (CML and AML) with BCR/ABL overexpression as well as for gastrointestinal stromal tumors (GIST) with c-KIT mutations (Bennasroune et al., 2004). Furthermore, TKIs have been developed for the treatment of advanced metastatic renal cell carcinoma (RCC; Pirrotta et al., 2011).

Demethylating agents such as azacytidine and decitabine have been approved for treatment of AML, MDS, and other hematopoietic disorders (Issa et al., 2005; Garcia-Manero, 2008).

Histone deacetylases inhibitors (HDACis) have been designed as direct antitumor agents with major anti-proliferative effects. Some HDACis have been approved for the treatment of cutaneous T cell lymphoma (CTCL) and recently also for peripheral T cell lymphoma. HDACis are further tested in clinical phase II and III trials for multiple entities such as cervical, breast, and ovarian cancer, sarcoma, lymphoma, myeloma, and leukemia (Marks and Xu, 2009; Khan and La Thangue, 2012; Ning et al., 2012).

In addition, the proteasome inhibitor bortezomib has been approved for treatment of MM and mantle cell lymphoma (Voorhees and Orlowski, 2006; Orlowski and Kuhn, 2008).

Current studies addressing the use of immune modulating pharmaceutics in different other malignant diseases are in progress. Interestingly, these new pharmaceuticals do not only show direct antitumor effects, but also impact cellular immunity. In this review, we sum up the recent knowledge of the influence each of these drugs has on natural killer (NK) cells.

Natural killer cells are a heterogeneous lymphocyte population with cytotoxic antitumor capacity and multiple immunoregulatory properties. They express a wide range of activating receptors [e.g. natural cytotoxicity receptors (NCRs), some killer-cell immunoglobulin-like receptors (KIRs), and NKG2D], as well as inhibitory receptors (some KIRs, co-receptor NKG2A) that regulate NK cell activation and tolerance (Vivier et al., 2011). In the last years, exiting data revealed that modulation of the balance between activating and inhibiting NK cell signals, sensitization of malignant cells to NK cell-mediated cytotoxicity, optimization of the cross-talk of NK cells with other immune cells and the improvement of adoptive NK cell therapeutic protocols may provide novel immunotherapeutic concepts for treatment of cancer (Terme et al., 2008).

## IMMUNOMODULATORY DRUGS

Thalidomide was described as anti-angiogenic, antitumor, and immune modulatory agent affecting many cell types. To overcome the devastating side effects, including congenital defects when used during pregnancy, novel classes of IMiDs^®^ have been developed (Bartlett et al., 2004a). The most important IMiDs^®^ are lenalidomide (CC-5013, Revlimid^®^) and pomalidomide (CC-4047, Actimid^®^) but further thalidomide analogs are under clinical trials (Bartlett et al., 2004a; Reddy et al., 2008).

Thalidomide and IMiDs^®^ were able to stimulate T cells to produce IFN-γ and IL-2 leading to NK cell activation (Davies et al., 2001; Hayashi et al., 2005). In addition, IMiDs^®^ also showed direct effects on NK cells. Recent preclinical data revealed an augmentation of NK cell percentages in pomalidomide- and lenalidomide-treated peripheral blood mononuclear cells (PBMCs) from healthy donors that was mediated by the phosphoinositide (PI)-3 kinase signaling pathway causing enhanced IL-2 transcription and secretion in T cells (Hayashi et al., 2005). Pomalidomide induced expression of CD69 accompanied by a more than twofold increase of IFN-γ production and enhanced killing of K562 tumor cells (Payvandi et al., 2005). Interestingly, NK cells were required but not sufficient for lysis of K562, meaning that cytotoxicity was also dependent on other cell populations within the PBMCs (Zhu et al., 2008). In line with that, PBMCs treated with thalidomide, lenalidomide, or pomalidomide gained an increased killing capacity of MM tumor cells that was lost upon NK cell depletion (Davies et al., 2001). In addition to enhanced cytotoxicity, lenalidomide, and pomalidomide also increased antibody-dependent cell cytotoxicity (ADCC) toward MM cells (Hayashi et al., 2005). It has further been confirmed that the effect of pomalidomide on cytotoxicity and ADCC was induced by IL-2-producing T cells. Although signaling molecules that could directly mediate NK cell cytotoxicity and ADCC like ERK or p38 mitogen-activated protein kinase (MAPK) were not activated by pomalidomide, both IMiDs^®^ were able to downregulate suppressor of cytokine signaling 1 (SOCS1) in NK, T, and NKT cells (Gorgun et al., 2010). Additionally, lenalidomide-treatment resulted in upregulation of CD16, CD40L, and LFA1 on NK cells thereby facilitating ADCC against MM cells (Tai et al., 2005). Recent work showed that lenalidomide does not affect NK cell proliferation among PBMCs but inhibits the production of IFN-γ in IL-12/IL-18 pre-activated, healthy donor-derived, purified NK cells. Furthermore, the NK cell phenotype was modified by enhancement of CD56 and decreased expression of KIRs and NKp46 (Dauguet et al., 2010). However, the reduced expression of receptors involved in NK cell-mediated killing was not sufficient to impair the cytolytic function of NK cells (Hayashi et al., 2005; Dauguet et al., 2010).

*In vivo*, administration of lenalidomide and pomalidomide resulted in a significant increase of NK cells in severe combined immunodeficient (SCID) mice with human lymphoma xenografts. The combination of IMiDs^®^ together with rituximab prolonged the median survival and was abrogated by NK cell depletion (Hernandez-Ilizaliturri et al., 2005). In Raji-bearing SCID mice, application of IMiDs^®^ led not only to NK cell expansion and activation but also to their migration into tumor beds. In this model, the effects of IMiDs^®^ seemed to be related to dendritic cells (DCs) and their secreted cytokines (Reddy et al., 2008).

Remarkably, in patients responding to thalidomide-therapy an increased percentage of CD56^+^ cells has been observed (Davies et al., 2001). This observation is in line with other studies reporting an enhanced number of NK cells in patients during IMiD^®^-treatment (Bartlett et al., 2004b). In a clinical study addressing the effects of lenalidomide on MM patients with relapse after allogeneic stem cell transplantation, the monitoring of immune cell subpopulations revealed an increase of T, Treg, and activated NKp44^+^ NK cells (Lioznov et al., 2010).

To sum it up, IMiDs^®^ mostly positively influence NK cell function, either direct or indirect via DC or T cell stimulation. Nevertheless, effects of IMiDs^®^ on NK cells should be further investigated in clinical immunomonitoring studies.

## TYROSINE KINASE INHIBITORS

Tyrosine kinase receptors are involved in multiple cellular processes and have a crucial role in tumor development and progression. The inhibitors of those receptors are called TKIs and belong to a broad range of drugs (Bennasroune et al., 2004). Important TKIs are imatinib (Givec^®^, Gleevec^®^) and nilotinib (Tasigna^®^) that specifically inhibit BCR/ABL, PDGFR, and c-KIT, as well as dasatinib (Sprycel^®^) that additionally targets the SRC kinase (Krusch and Salih, 2011). Further multi-target TKIs like sunitinib (Sutent^®^) and sorafenib (Nexavar^®^) have been developed inhibiting various tyrosine kinases (Pirrotta et al., 2011).

Recent *in vitro* and *in vivo* studies indicated both direct inhibitory effects on immune cells including T and NK cells and indirect activatory or inhibitory effects on NK cell function via modification of markers on tumor cells caused by TKI-treatment (Seggewiss et al., 2005; Chen et al., 2008; Schade et al., 2008; Weichsel et al., 2008; Fraser et al., 2009). On side of the tumor, a direct control of the expression of the NKG2D ligands (NKG2DLs) MHC class I-related chain molecules (MIC)A/B by BCR/ABL has been shown and was reduced by different TKIs leading to decreased NK cell-mediated cytotoxicity and IFN-γ production (Boissel et al., 2006; Salih et al., 2010). A similar effect was shown after imatinib-treatment of a leukemic cell line transfected with high levels of BCR/ABL representing an ideal NK cell target. Imatinib led to diminished killing that was accompanied by decreased ICAM-1 expression on target cells and was most likely due to reduced formation of NK cell/target immunological synapses (Baron et al., 2002; Cebo et al., 2006). On the NK cell effector side, direct exposure of human NK cells with pharmacological doses of imatinib had no impact on NK cytotoxicity or cytokine production, whereas nilotinib negatively influenced cytokine production and dasatinib additionally abrogated cytotoxicity *in vitro*. The direct modulation of NK cells by dasatinib was apparently based on its impact on signaling cascades preventing phosphorylation of PI-3 kinase and ERK1/2 (Salih et al., 2010). Interestingly, inhibition by dasatinib seemed to be reversible as washing NK cells mainly restored cytotoxicity (Blake et al., 2008; Hassold et al., 2012).

Further, in a murine model NK cell activation could be induced by imatinib-treated DC *in vitro* and *in vivo* (Borg et al., 2004). The positive, most likely NK cell-dependent, antitumor effect of imatinib was further augmented by IL-2 in another murine model (Taieb et al., 2006). Other data showed, that frequencies of NK cells were not altered by imatinib-treatment in mice (Balachandran et al., 2011).

In contrary to the TKIs described so far, treatment of tumor cells with the multi-kinase inhibitors sorafenib and sunitinib increased their susceptibility for cytolysis by NK cells. Treatment of a hepatocellular carcinoma cell (HCC) line with sorafenib did not affect HLA class I expression but increased membrane-bound MICA and decreased soluble MICA resulting in enhanced NK cell-mediated cytotoxicity. Sorafenib led to a decline of the metalloprotease ADAM9 that is usually upregulated in human HCC resulting in MICA shedding (Kohga et al., 2010). Also, incubation of a nasopharyngeal carcinoma cell line with sunitinib increased the expression of NKG2DL better than sorafenib leading to a higher NK cell-mediated cytotoxicity (Huang et al., 2011). On the other side, in line with the other TKIs mentioned before, pharmacological concentrations of sorafenib but not sunitinib reduced cytotoxicity and cytokine production of resting and IL-2-activated NK cells *in vitro* by impaired granule mobilization apparently due to diminished phosphorylation of ERK1/2 and PI-3 kinase. Notably, sunitinib only altered cytotoxicity and cytokine production when added in high doses which were not reached in patients (Krusch et al., 2009).

In immunomonitoring analysis, NK cell percentages did not differ between imatinib-treated Philadelphia chromosome positive ALL patients and healthy donors (Maggio et al., 2011). In CML patients, the NK cell percentages were decreased at diagnosis and did not recover during imatinib therapy. This was accompanied by reduced degranulation response to tumor cells (Chen et al., 2012). Another study compared NK cell numbers of patients who received imatinib with complete molecular response for more than 2 years, patients that stopped therapy, and healthy donors. Interestingly, NK cell numbers were significantly increased in patients that stopped therapy. Of note, increasing cell numbers correlated with increased NK cell activity (Ohyashiki et al., 2012). During imatinib therapy of GIST patients an increase of INF-γ production by NK cells was observed and correlated with a positive therapy response (Borg et al., 2004). Although GIST patients displayed less NKp30^+^ NK cells and fewer NKp30-dependent lytic potential, both were at least partially restored during imatinib therapy. On the other hand, NKG2D showed a normal expression on NK cells in GIST patients, but nevertheless imatinib increased NKG2D-dependent cytotoxicity. Additionally, after 2 months of therapy, imatinib led to increased IFN-γ production of patient-derived NK cells after restimulation with IL-2 or DCs *in vitro* (Menard et al., 2009). In contrast to the observation of NK cell suppression by dasatinib *in vitro* as well as in murine models, where dasatinib-treatment led to reduced lysis of tumor cells, some dasatinib-treated patients showed an increased number of NK large granular lymphocytes associated with improved leukemic control and prolonged survival (Fraser et al., 2009; Kim et al., 2009; Mustjoki et al., 2009; Kreutzman et al., 2010; Tanaka et al., 2012). Regarding the effects of sunitinib in clinical settings, there was no significant change concerning NK cell numbers measured in 43 metastatic clear cell renal cancer patients (Powles et al., 2011). Recently, a link between KIR genotypes and therapy response to TKIs has been assessed. Interestingly, the lower BCR/ABL1 transcript levels in peripheral blood of 86 first-line dasatinib-treated CML patients after 12 months were associated with the absence of three inhibitory KIRs and a similar trend was visible for two activating KIRs including KIR2DS1 (Kreutzman et al., 2012). KIR genotyping in 174 CML patients on first-line therapy with imatinib showed that the expression of KIR2DS1 was accompanied by a significantly lower 2-year probability to achieve cytogenetic response (Marin et al., 2012). In contrast, another study showed no significant impact of the KIR genotype on clinical outcome in 130 CML patients (Ali et al., 2012). However, missing KIR ligand KIR/HLA genotype combination might be a prognostic factor as in the case of increased survival in neuroblastoma patients treated with anti-GD2 antibody (Tarek et al., 2012).

Altogether, TKIs display various direct and indirect effects on NK cells and function. Dasatinib, imatinib, and nilotinib showed a negative influence on NK cell ligands on tumor cells whereas the effect of sorafenib and sunitinib revealed an obverse effect. With exception of imatinib the direct effects on NK cells seem to be mostly inhibitory and should be further investigated.

## DEMETHYLATING AGENTS

DNA hypermethylation followed by silencing of tumor-suppressor genes is supposed to play an important role in leukemogenesis. Demethylating agents like azacytidine (5-azacytidine, Vidaza^®^) and decitabine (5-aza-2^′^-deoxycytidine, 5-aza-2^′^-dc, Dacogen^®^) are cytidine analogs irreversibly inhibiting the DNA methyltransferase and in addition induce genome-wide damages in a dose-dependent manner. Demethylating agents have already revealed their direct effects on malignant cells (Christman, 2002; Issa et al., 2005; Stone et al., 2009).

It was shown that azacytidine and decitabine alone or combined with other drugs induced expression of the NKG2DLs MICA/B or UL16-binding proteins (ULBP) on various tumor cell lines (Rohner et al., 2007; Tang et al., 2008; Wu et al., 2009; Schmiedel et al., 2011). Interactions of NKG2D with chemically induced MICB significantly enhanced the killing capacity of a NK cell line upon pre-treatment with decitabine, most likely due to DNA damage and promotor DNA demethylation (Gasser et al., 2005; Tang et al., 2008). NK cell-dependent antitumor activity was also increased upon treatment of patient-derived primary AML blasts with a combination of demethylating agents and other differentiation-promoting drugs (Rohner et al., 2007). Demethylating agents do not only affect tumor cells, they additionally influence gene expression in effector cells. NK cell lines, clones and developing NK cells all exhibiting different KIR genotypes, displayed a stable transcription of KIRs after treatment with decitabine (Santourlidis et al., 2002; Chan et al., 2005). In contrast to the augmented tumor susceptibility to lysis upon azanucleoside-treatment, it has been reported that azacytidine induced expression of inhibitory KIRs and led to impaired granzyme B and perforin release resulting in limited cytolytic function (Gao et al., 2009).

An important comparative analysis of azacytidine and decitabine revealed different impacts on NK cell cytotoxicity. While azacytidine-treated PBMC-derived NK cells displayed a diminished cytotoxicity, decitabine-treated cells showed the opposite and strongly dose-dependent effect. In line with the impact on cytotoxicity, azacytidine led to decreased and decitabine to increased IFN-γ production by NK cells upon stimulation with tumor cells or IL-2. The expression of activating receptors was only influenced by azacytidine resulting in slight downregulation of CD16, NKG2D, and NKp30 on NK cells. Of note, the relatively short exposure time to demethylating agents in the described *in vitro* setting did neither alter the KIR profile of NK cells nor the NKG2DL expression of target cells that has been described previously. Altogether, the inhibitory effect of azacytidine seemed to be caused by impaired mRNA synthesis, whereas decitabine increased the responsiveness to activation stimuli resulting in enhanced gene transcription (Schmiedel et al., 2011).

However, one should be aware that these studies have mainly been performed *in vitro* and so far analysis of NK cell phenotype and function from patients treated with demethylating agents are pending.

## HISTONE DEACETYLASES INHIBITORS

Histone deacetylases (HDACs) play an important role in the epigenetic modulation of gene expression affecting cell-cycle arrest, differentiation and apoptosis in all nucleated cells. Mutations in genes encoding HDACs and alterations in HDAC expression have been found in many types of cancer. Therefore, inhibition of HDACs seems to be a good point of attack in tumor therapy (Glozak and Seto, 2007). Mentionable HDACis are sodium valproate (VPA), trichostatin A (TSA), sodium butyrate, vorinostat (Zolinza^®^), romidepsin (Istodax^®^), and chidamide (CS055/HBI-8000).

Microarray analysis revealed that VPA augmented MICA/B expression on HCC cells leading to elevated lysis by NK cells (Armeanu et al., 2005). A similar effect could be shown *in vitro* in treated leukemic blasts from AML patients that enhanced MICA/B and ULBP1 expression, thereby increasing cytolysis by KIR–HLA class I-mismatched cells (Diermayr et al., 2008). Recently, a similar effect was shown on a B-ALL cell line and a proportion of primary B-ALL samples. Those tumor cells upregulated NKG2DL and displayed an enhanced, NKG2D-dependent degranulation of NK cells (Jardine et al., 2012). TSA-treated leukemic cell lines and patient-derived leukemic cells also increased MICA/B expression via histone acetylation of promotors and mediated NK cell cytotoxicity (Kato et al., 2007). These NK cell-activating effects could also be demonstrated for vorinostat and sodium butyrate. This study further showed a cell-dependent involvement of cytotoxicity mediated via DNAX accessory molecule-1 (DNAM-1) and NCRs (Schmudde et al., 2008). Noteworthy, romidepsin-dependent upregulation of MICA/B on cancer cells showed an essential involvement of the glycogen synthase kinase-3, whereas the sodium butyrate-induced upregulation was influenced by increased binding of Sp1 together with heat shock transcription factor 1 to the MICA/B promotor (Skov et al., 2005; Zhang et al., 2009). On the effector side, one of the newest HDACi, chidamide, led to enhanced lysis of K562 tumor cells *in vitro* by increased expression of proteins involved in NK cell functions like CD16, NKG2D, and granzyme A. Gene expression studies were performed and a time-dependent induction of NK cell receptors (CD16, NKG2D, and KLRG1), cytotoxic enzymes (granzyme and perforin), and molecules important for apoptosis (FASLG) was observed (Ning et al., 2012). On the other hand, there is increasing evidence that HDACis can also inhibit cytotoxicity even of activated NK cells. In one study, VPA- and vorinostat-induced inhibition was accompanied by diminished expression of NKp46 and NKp30 and reduced granule exocytosis (Ogbomo et al., 2007). Recent data on the effect of vorinostat on NK cells from healthy donors and patients with CTCL also demonstrated a suppression of cytotoxicity *in vitro* (Anshelevich et al., 2010). A further study distinguished between activated and resting NK cells and revealed a downregulation of NKG2D and NKp46 on resting, VPA-, TSA-, or sodium butyrate-treated, and an additional downregulation of NKp44 and CD25 on activated, TSA-treated NK cells. These HDACis also significantly impaired IFN-γ production of NK cells (Rossi et al., 2012). VPA decreased IFN-γ expression of cytokine-pre-treated NK cells on mRNA and protein levels that was associated by decreased phosphorylation of STAT5 and attenuated expression of T-bet. Valproic acids further reduced expression of perforin and granzyme B, whereas only the transcript levels and not the protein levels of granzyme A and Fas ligand were affected (Alvarez-Breckenridge et al., 2012).

In a murine tumor model, treatment with TSA showed reduced expression of NK1.1, NKG2D, and NKp46 accompanied with diminished IFN-γ production after *ex vivo* cytokine stimulation (Rossi et al., 2012). Furthermore, immunomonitoring performed on romidepsin-treated CTCL patients showed suppression of cellular immune functions, including NK cell cytotoxicity (Kelly-Sell et al., 2012).

In sum, these data show not only the promising effect on tumor sensitization to NK cell cytotoxicity, but also a potential negative impact on antitumor NK cell activity by different HDACis with exception of new agents like chidamide. However, further evaluation in clinical studies is needed.

## PROTEASOME INHIBITORS

Proteasomes belong to the intracellular machinery responsible for protein degradation. Inhibitors of proteasomes can directly inhibit tumor growth or sensitize cells to apoptotic effects of other agents. The mechanisms by which bortezomib (Velcade^®^), an important proteasome inhibitor, acts, may differ dependent on tumor targets and still have to be further examined (Voorhees and Orlowski, 2006).

In general, tumor-treatment with proteasome inhibitors seemed to sensitize various tumor cells and cell lines such as breast cancer, melanoma, RCC, CML, and others to NK cell-mediated lysis (Lundqvist et al., 2006, 2010; Ames et al., 2009; Yong et al., 2009). First studies showed that bortezomib led to expression of death receptor DR5 on RCC cells that correlated with susceptibility toward NK cells (Lundqvist et al., 2006). Further *in vitro* analysis indicated that bortezomib-treated tumors had higher TRAIL-induced caspase 8 activity (Lundqvist et al., 2009). Additionally, bortezomib and other tested proteasome inhibitors with distinct mechanisms also strongly increased NKG2DL expression such as ULBP in head and neck squamous cell carcinoma cells and other cell lines (Vales-Gomez et al., 2008; Butler et al., 2009). This could be recently confirmed as bortezomib augmented NKG2DL on a B-ALL cell line and a proportion of primary B-ALL samples. All treated cells displayed an enhanced degranulation of NK cells mediated by NKG2D signaling (Jardine et al., 2012). In line with that *in vitro* treated primary plasma cells upregulated DNAM-1 and NKG2DL accompanied with increased degranulation of NK cells (Soriani et al., 2009). On the contrary, other studies described a bortezomib-dependent downregulation of HLA class I on MM cell lines and patient-derived MM cells in a dose- and time-dependent manner. This effect was also observed on MM cells derived from patients treated with bortezomib (Shi et al., 2008). However, besides this augmentation of cytotoxicity, pro-apoptotic effects of bortezomib on PBMC-derived, resting NK cells have been reported. This effect might be due to oxidative stress as glutathione significantly abolished total NK cell apoptosis by preventing loss of mitochondrial membrane potential. Furthermore, bortezomib-treated NK cells downregulated NKp46 leading to diminished NKp46-mediated NK cell activity (Wang et al., 2009). Besides, NK cytotoxicity could also be diminished by downregulation of TRAIL via inhibition of NFκB on IL-2-activated NK cells upon bortezomib-treatment *in vitro* (Feng et al., 2010).

Murine studies showed a NK cell-sensitizing effect of bortezomib-treated renal tumors to both perforin/granzyme and TRAIL-mediated lysis *in vitro*. In addition, bortezomib-treatment combined with Treg depletion significantly enhanced tumor lytic capacity of adoptively transferred autologous NK cells *in vivo* (Lundqvist et al., 2009). Of note, additional work in this mouse model revealed that bortezomib-treated tumors underlying enhanced NK cell killing paradoxically developed resistance to antigen-specific T cells that was probably due to changes in proteasomal processing and presentation of antigens (Lundqvist et al., 2010). In another *in vitro* mouse model, sensitization of tumor cells with bortezomib led to lysis that was mediated by enhanced DR5 and Fas expression accompanied with downregulation of MHC class I. The authors could show in a murine tumor model that bortezomib-treatment in bone marrow transplantation together with NK cell immunotherapy increased survival (Hallett et al., 2008).

Natural killer cells from RCC patients showed an increased killing against autologous tumor cell lines after *ex vivo* sensitization with bortezomib (Lundqvist et al., 2006). A first phase I trial showed safety in adoptive transfer of activated NK cells. This protocol was combined with Treg depletion and bortezomib-treatment for tumor sensitization prior to adoptive NK cell transfer into patients with advanced stage of cancer (Lundqvist et al., 2011).

Although tumor cells displayed an increased NK cell susceptibility upon stimulation with proteasome inhibitors, the effect on NK cells seemed to be contrary and should be closer examined. In addition, further clinical phase II and III studies are needed to address the specific tumor entity and combination therapy that might promise best efficacy of the combination of tumor sensitization with proteasome inhibitors and donor lymphocyte infusion of NK cells (NK-DLI).

## CONCLUDING REMARKS AND OUTLOOK

In summary, the heterogeneous impact on NK cell function reported for the different immune modulating substances, referred in this review, is mediated by either indirect or direct effects on NK and tumor cells and is further depending on the tumor entity, timing, and dosing of the different pharmaceutics (**Table 1**).

**Table 1:** Overview on the impact of immunomodulating agents on NK cells.

Drug	Indirect effect on NK cells	Direct effect on NK cells
**IMiDs^®^**
Thalidomide	•T cell stimulation leading to increased ADCC and cytotoxicity of NK cells toward K562 and MM tumor cells (Hayashi et al., 2005)	•Increased NK cell numbers after therapy in MM patients (Davies et al., 2001) •Increased cytotoxicity toward MM tumor cells (Davies et al., 2001)
Lenalidomide	•Increased NK cell numbers and activation in Raji-bearing SCID mice via stimulation of DCs and their cytokines (Reddy et al., 2008) •T cell stimulation leading to increased ADCC and cytotoxicity of NK cells toward K562 and MM tumor cells (Hayashi et al., 2005)	•No effect on proliferation of NK cells *in vitro* (Dauguet et al., 2010) •Increased NK cell numbers in metastatic MM and other advanced cancer patient (Bartlett et al., 2004b) •Increased numbers of NKp44^+^ NK cells in MM patients with relapse (Lioznov et al., 2010) •Upregulation of CD56, CD16, CD40L, and LFA1 (Tai et al., 2005; Dauguet et al., 2010) •Downregulation of SOCS1, NKAT2, NKB1, CD158ah, and NKp46 (Dauguet et al., 2010; Gorgun et al., 2010) •Increased cytotoxicity toward MM tumor cells (Davies et al., 2001) •Inhibition of IFN-γ production in activated NK cells (Dauguet et al., 2010)
Pomalidomide	•Increased NK cell numbers and activation in Raji-bearing SCID mice via stimulation by DCs and their cytokines (Reddy et al., 2008) •T cell stimulation leading to increased ADCC and cytotoxicity of NK cells toward K562 and MM tumor cells (Hayashi et al., 2005) •Upregulation of CD69 (Payvandi et al., 2005) •Increased IFN-γ production (Payvandi et al., 2005)	•Downregulation of SOCS1 (Gorgun et al., 2010) •Increased cytotoxicity toward MM and K562 tumor cells (Davies et al., 2001; Payvandi et al., 2005)
**TKIs**
Imatinib	•Downregulation of NKG2DL on K562 and CML tumor cells leading to reduced cytotoxicity and IFN-γ production (Salih et al., 2010) •Downregulation of ICMA-1 on BCR/ABL-transfected leukemic tumor cells accompanied by decreased cytotoxicity (Baron et al., 2002) •DC stimulation leading to increased NK cell activity in human and mice (Borg et al., 2004)	•Increased NK cell numbers post therapy (Ohyashiki et al., 2012) •No impact on cytotoxicity and cytokine production *in vitro* (Salih et al., 2010) •Increased NKp30- and NKG2D-dependent lysis and IFN-γ production in GIST-patients (Menard et al., 2009) •Increased INF-γ production in GIST patients correlated with clinical outcome (Borg et al., 2004)
Nilotinib	•Downregulation of NKG2DL on K562 and CML tumor cells leading to reduced cytotoxicity and IFN-γ production (Salih et al., 2010)	•Inhibition of cytokine production but no effect on cytotoxicity (Salih et al., 2010)
Dasatinib	•Downregulation of NKG2DL on K562 and CML tumor cells leading to reduced cytotoxicity and IFN-γ production (Salih et al., 2010)	•Inhibition of cytotoxicity in mice (Fraser et al., 2009) •Inhibition of cytotoxicity toward K562 and cytokine production (Salih et al., 2010)
Sorafenib	•Increase of MICA on HCC tumor cells and decrease of soluble MICA leading to enhanced cytotoxicity (Kohga et al., 2010) •Increased expression of NKG2DL on nasopharyngeal carcinoma cells leading to enhanced cytotoxicity (Huang et al., 2011)	•Inhibition of cytotoxicity and cytokine production (Krusch et al., 2009)
Sunitinib	•Increased expression of NKG2DL leading to enhanced cytotoxicity (Huang et al., 2011)	•Inhibition of cytotoxicity and cytokine production only in unphysical high doses (Krusch et al., 2009)
**Demethylating agents**
Azacytidine	•Expression of NKG2DL on K562 and Raji tumor cell (Schmiedel et al., 2011)	•Induction of inhibitory KIRs resulting in decreased cytotoxicity (Gao et al., 2009) •Downregulation of CD16, NKG2D, and NKp30 (Schmiedel et al., 2011) •Inhibition of cytotoxicity toward K562 and Raji tumor cells and cytokine production (Schmiedel et al., 2011)
Decitabine	•Expression of NKG2DL on various tumor cell lines including K562 and Raji cells leading to enhanced cytotoxicity (Rohner et al., 2007; Tang et al., 2008; Wu et al., 2009; Schmiedel et al., 2011)	•General induction of KIRs (Santourlidis et al., 2002) •Increasing of cytotoxicity toward K562 and Raji tumor cells and cytokine production (Schmiedel et al., 2011)
**HDACis**
VPA	•Expression NKG2DL on HCC and AML tumor cells leading to enhanced cytotoxicity (Armeanu et al., 2005; Diermayr et al., 2008) •Upregulation of NKG2DL on B-ALL tumor cells leading to enhanced degranulation (Jardine et al., 2012)	•Downregulation of NKG2D, NKp46, and NKp30 (Ogbomo et al., 2007; Rossi et al., 2012) •Inhibition of cytotoxicity toward various tumor cells (Ogbomo et al., 2007; Alvarez-Breckenridge et al., 2012) •Inhibition of cytokine production (Alvarez-Breckenridge et al., 2012; Rossi et al., 2012)
TSA	•Increased NKG2DL expression on various leukemic tumor cells leading to enhanced cytotoxicity (Kato et al., 2007)	•Downregulation of NKG2D and NKp46 on resting and additionally NKp44 and CD25 on activated NK cells (Rossi et al., 2012) •Downregulation of NK1.1, NKG2D, and NKp46 in mice (Rossi et al., 2012) •Inhibition (Rossi et al., 2012)
Vorinostat	•Sensitizing various tumor cells toward cytotoxicity (Schmudde et al., 2008)	•Downregulation of NKp46 and NKp30 (Ogbomo et al., 2007) •Inhibition of cytotoxicity toward various tumor cells (Ogbomo et al., 2007; Anshelevich et al., 2010) •Inhibition of cytokine production (Rossi et al., 2012)
Sodium butyrate	•Sensitizing various tumor cells toward cytotoxicity (Schmudde et al., 2008)	•Downregulation of NKG2D and NKp46 on resting NK cells (Rossi et al., 2012) •Inhibition of cytokine production (Rossi et al., 2012)
Romidepsin	•Upregulation of NKGDL on tumor cells (Skov et al., 2005)	•Inhibition (Kelly-Sell et al., 2012)
Chidamide		•Upregulation of CD16, NKG2D, granzyme, perforin, KLRG1, and FASLG (Ning et al., 2012). •Increased cytotoxicity toward K562 tumor cells (Ning et al., 2012)
**Proteasome inhibitors**
Bortezomib	•Induction of DR5 on renal cell carcinoma leading to enhanced susceptibility (Lundqvist et al., 2006) •Upregulation of NKG2DL on diverse cell lines and head and neck squamous cell carcinoma cells leading to enhanced susceptibility (Vales-Gomez et al., 2008; Butler et al., 2009) • Increased caspase 8 activity leading to enhanced susceptibility toward murine and human tumors (Lundqvist et al., 2009) •Downregulation of HLA class 1 on human MM and MHC class I on murine leukemic tumor cells (Hallett et al., 2008; Shi et al., 2008) •Upregulation of DNAM-1 and NKG2DL in primary plasma cells and NKG2DL on B-ALL tumor cells leading to increased degranulation of NK cells (Soriani et al., 2009; Jardine et al., 2012)	•Pro-apoptotic effect on resting NK cells (Wang et al., 2009) •Downregulation of NKp46 resulting in diminished activity (Wang et al., 2009) •Inhibition of cytotoxicity of activated NK cells toward MM tumor cells (Feng et al., 2010)

The majority of IMiDs^®^ predominantly mediates immune stimulation positively influencing NK cell-mediated antitumor capacity. TKIs, HDACis, and proteasome inhibitors exhibit various effects. They display mostly negative impacts on NK cell function although treatment of tumor cells may result in contradictory results by sensitization to apoptosis-induction. Exceptions were the new HDACi chidamide and the TKI imatinib that showed a positive effect on NK cell activation. Both of the demethylating agents described exhibit opposite effects on NK cell function.

For better understanding of the underlying mechanisms, unambiguous data on NK cell function from preclinical and clinical studies are still needed. In addition to the complex balance of both tumor and NK cells, there are multiple effects on the other players of the immune system such as antigen presenting cells and cytolytic T cells that regulate the outcoming immune effects.

Finally, an ideal therapeutic concept for cancer patients might combine inhibitors of immunosuppression with tumor sensitizers and cellular therapy protocols such as adoptive NK cell transfer. This review underlines the urgent need of further immunomonitoring studies that should address functional aspects of immune cells on autologous patient-derived tumor cells under therapy with immune modulating agents. Growing knowledge from such studies will allow the right choice of the ideal immune modulators at the optimal dosing and timing to obtain the best possible outcome for the patients.

## Conflict of Interest Statement

The authors declare that the research was conducted in the absence of any commercial or financial relationships that could be construed as a potential conflict of interest.

## Abbreviations

ADCC: antibody-dependent cell cytotoxicity
AML: acute myeloid leukemia
CLL: chronic lymphatic leukemia
CML: chronic myeloid leukemia
CTCL: cutaneous T cell lymphoma
DC: dendritic cell
DLI: donor lymphocyte infusion
DNAM-1: DNAX accessory molecule-1
GIST: gastrointestinal stromal tumors
HCC: hepatocellular carcinoma cell
HDAC: histone deacetylase
HDACi: histone deacetylase inhibitor
IMiD^®^: immunomodulatory drug
KIR: killer-cell immunoglobulin-like receptors
MAPK: mitogen-activated protein kinase
MDS: myelodysplastic syndrome
MIC: MHC class I-related chain molecules
MM: multiple myeloma
NCR: natural cytotoxicity receptor
NK: natural killer
NKG2DL: NKG2D ligand
PBMC: peripheral blood mononuclear cells
PDGFR: platelet-derived growth factor receptor
PI: phosphoinositide
RCC: renal cell carcinoma
SCID: severe combined immunodeficient
SOCS1: suppressor of cytokine signaling 1
TCL: T cell lymphoma
TKI: tyrosine kinase inhibitor
TSA: trichostatin A
VEGFR: vascular endothelial growth factor receptor
VPA: sodium valproate.
